# Antisocial Disorders in Adolescence and Youth, According to Structural, Emotional, and Cognitive Transdiagnostic Variables: A Systematic Review

**DOI:** 10.3390/ijerph17093036

**Published:** 2020-04-27

**Authors:** Ronald Toro, Juan García-García, Flor Zaldívar-Basurto

**Affiliations:** 1 Psychology Department, University of Almería, 04120 Almería, Spain; tororonald@gmail.com; 2 Psychology Department, Health Research Center, University of Almería, 04120 Almería, Spain; flor@ual.es

**Keywords:** transdiagnostic, antisocial, externalizing, criminal behavior, juvenile delinquency, social deviance

## Abstract

Transdiagnostic causal variables have been identified that have allowed understanding the origin and maintenance of psychopathologies in parsimonious explanatory models of antisocial disorders. However, it is necessary to systematize the information published in the last decade. The aim of the study was to identify through a systematic review, the structural, emotional and cognitive transdiagnostic variables in antisocial disorders of adolescence and youth. Recommendations for systematic reviews and meta-extraction and analysis of information according to the Preferred Reporting Items for Systematic Review and Meta-Analyses (PRISMA), the Cochrane Collaboration and Campbell were followed. We found 19 articles from 110 reviewed documents. The results indicated that at a structural level there is a general psychopathological factor (psychopathy or externalizing), non-emotional callousness and impulsivity from behavioral inhibition and activation systems, and negative affect traits as base structures. In the emotional level, the study found a risk component from emotional dysregulation and experiential avoidance. In the cognitive level, a key role of anger-rumination and violent ideation as explanatory variables of antisocial disorders. We concluded that the interaction of these identified variables makes it possible to generate an evidence-based transdiagnostic model.

## 1. Introduction

**Figure 1:**
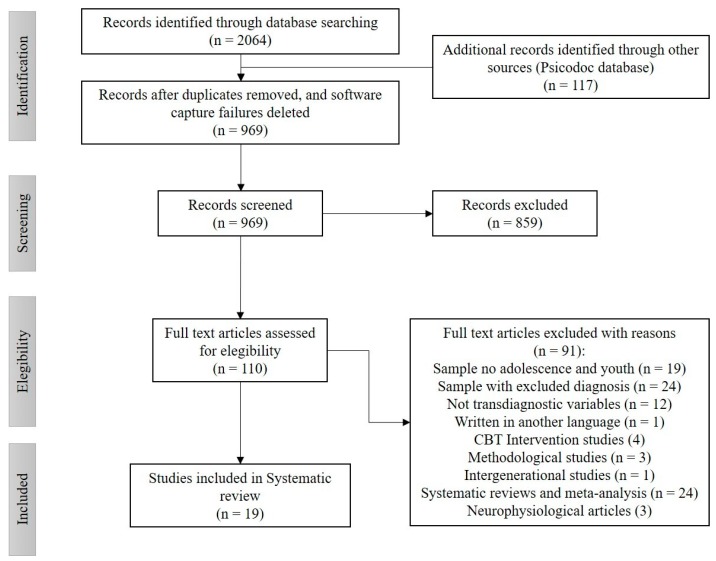
PRISMA model of systematic review study identification and inclusion.

Antisocial disorders (ADs) are characterized by alterations in impulse control, emotional dysregulation and behavioral problems, manifested in behaviors that violate the rights of others such as delinquency, property damage, aggression, and conflicts with accepted social norms and authority figures [1,2]. ADs have been analyzed from a dimensional spectrum made up of a group of psychopathologies, which are organized according to their common etiologies and symptoms, resulting in the externalizing spectrum [3]. In DSM 5 [2], they appear in the group of ADs of childhood, adolescence, and adulthood, which includes conduct disorders (including specification: with limited pro-social emotions), intermittent explosive disorder and antisocial personality disorder, in addition to antisocial behavior in the child or adolescent located in other disorders associated with disruptive or impulsive behavior.

It is possible to develop models that guide the understanding of the origin and maintenance, from the common variables called transdiagnostic by using a spectrum as an indicator of AD in psychopathology research, following a dimensional perspective of the processes and factors underlying the different disorders. The transdiagnostic model (TM) “consists of understanding mental disorders based on a range of etiopathogenic cognitive and behavioral processes that are the cause or maintain most of the mental disorders or consistent groups of mental disorders” ([4], p. 187).

This model has developed research based on the symptoms and processes underlying mental illness, following a limited range of common symptoms, or diagnoses with multiple processes, or others, with single universal and multiple processes [5]. The transdiagnostic approach to multiple processes seeks the least number of key mechanisms with the greatest explanatory power in the understanding of different comorbid externalizing psychopathological disorders [6]. To this end, variables have been suggested that have generated new lines of research and intervention in AD, such as cognitive rumination, emotional dysregulation (ED), behavioral activation and inhibition systems, among others. However, this research around a transdiagnostic model is still limited in its development and scope; although the studies still have small samples and the available intervention protocols are scarce, more research is needed on its efficacy in the different comorbid problems—especially antisocial ones since they have focused on internalizing ones such as anxiety and depression [7].

In the adolescent and young adult population, an empirically reviewed TM requires a stance that allows for the integration of various levels of analysis that has an impact on efforts focused on the promotion and prevention of these problems [8]. When identifying the interactions between the variables of vulnerability that increase the risk of AD at these ages, whether emotional, cognitive, biological, and contextual, it is possible to propose a TM that allows for the prediction of future incremental risk in symptomatic severity. These transdiagnostic approaches are found in studies on impulsivity and addictive disorders within the framework of the research domain criteria (RDoC), which seeks to develop a multidimensional explanatory model at the bio-behavioral level ranging from the molecular, genetic to the behavioral [9]. Among the advances for a TM are studies on inhibitory control and reward systems as motivational mechanisms at the structural level.

In turn, reports with a transdiagnostic scope from the RDoC framework, have stood out for presenting genetic and psychophysiological variables and in models of traits oriented towards broad range factors such as externalizing or psychopathic factors. For example, recent studies have reported that adolescence and adulthood are characterized by significant changes in gray matter structure - volume - and genes associated with reward-seeking (*DRD2, DRD4, DAT1, OPRM1, COMT*). It leads to increases in impulsivity, especially in people with heavy drinking problems [10]. Increases derived from the interaction of genes and development explain variations in accelerated sensorimotor maturation for the cortex (high and low executive control, reward evaluation, and working memory) [10,11]. In other studies, psychopathy has generated advances in the understanding of the spectrum of AD, which is characterized by difficulties in establishing genuine interpersonal relationships, superficial affection, and chronic antisocial behavior [12]. Psychopathy also has been considered a continuum from primary psychopathy (emotional deficit) to secondary psychopathy (pathological level) with uninhibited profiles and interpersonal problems [13], aggressive behavior with anger dysregulation, and emotional withdrawal [14].

However, despite extant evidence suggesting a strong association between various variables and the externalizing problems, it is necessary to go deeper into these transdiagnostic variables. It is imperative to analyze the interaction between transdiagnostic variables at the different levels, as suggested by the RDoC framework, allowing their integration into an explanatory TM of the AD. Thus, this study was aimed at identifying, through a systematic review, the transdiagnostic variables and their interaction at the structural, emotional, and cognitive levels in the studies comprising the spectrum of AD in adolescence and youth.

## 2. Materials and Methods

Recommendations for the development of systematic reviews and meta-extraction and analysis of the information consulted were followed following the Preferred Reporting Items for Systematic Review and Meta-Analyses (PRISMA), the Cochrane Collaboration, and the Campbell Collaboration as a registry protocol [15,16].

### 2.1. Eligibility Criteria and Inclusion of Articles

The articles consulted were included if they met the criteria as corresponding to (a) a quantitative study with cross-sectional, longitudinal, correlational or experimental designs, with multivariate statistical analysis; (b) participants who meet the age requirements for adolescence and youth, following the classification of the World Health Organization [17], in which the second decade corresponds to the transition to adulthood, from puberty to full youth in three stages. Studies which included participants with older adults or children not considered in adolescence were excluded; (c) Instruments that included antisocial psychopathologies such as checklists, self-reports, clinical interviews, specific tests, and psychophysiological measures, as well as those that measured transdiagnostic variables; likewise, (d) there were no restrictions in the process of comparing the characteristics of the studies, and it was ensured that (e) the results (Outcomes) reported contributions to the development of a TM of the AD spectrum.

### 2.2. Information Sources

Table 1 describes the databases consulted, and the descriptors entered in the meta-searches. Similarly, Figure 1 shows the search process carried out between January and February 2020. The search parameters were documents written in English published in the period between 2010 and 2020, in which the RDoC model and its transdiagnostic integration with psychopathology was established as a starting point [9,18].

### 2.3. Search

The search process was carried out following the combinations of the descriptors set out in Table 1, the documents initially selected were downloaded as reference lists in the Mendeley Desktop software version 1.17.13 to facilitate their organization and download in an Excel matrix as a list of references including the abstract.

### 2.4. Selection of Studies

The three researchers determined the assessment of descriptors and databases. The first search was conducted by the first researcher (RT) and was shared as reference lists and abstracts with the other two researchers (JG and FZ). The initial selection of relevant documents for the study (*n* = 2181), was submitted by RT for evaluation by marking their nominal eligibility (0 = no, 1 = yes), rejection or possible choice (11.35%, 110 documents out of 969). Subsequently, JG and FZ prepared the eligibility review and issued their observations on 11.35% of the selected papers. To assess expert agreement, Fleiss’s kappa statistic was calculated. This statistic is designed to estimate multiple agreements for all researcher conjoint. Fleiss’s kappa generates a number representing the proportionate agreement adjusted for a chance where the value 0 means no agreement beyond chance, and the value 1 means perfect agreement [19]. The researchers’ level of agreement scores was then analyzed, Fleiss Kappa = 0.80; IC95% [0.70–0.91] showed that there was good agreement (calculated by JG). Nonetheless, the final agreement was resolved with consensus from all the authors.

### 2.5. Data Extraction Process

We obtained data from the 110 full-text articles included after review by the first investigator (RT) and verification by the two jurors (JG and then FZ). Once the scores were reviewed in agreement, the data was extracted with the final full-text articles (*n* = 19, 17.27%, of *n* = 110, 100%). Which went on to the systematic review in which the authors, countries, sample(s), ages, sexes, AD evaluated, measurement instruments, the transdiagnostic variables identified, and the main findings were extracted.

### 2.6. Bias Risk

For this study, we carried out the risk of bias by establishing criteria for reviewing the methodological quality of each full-text article analyzed. We used the items of the scoring algorithm of the device for assessing the methodological quality of intervention research [DIAD] [20] as a basis. When conducting the assessment, we took into account the type of study, sample size, description of the selection of participants (adolescents and young people), reliable and valid measures and instruments, use of transdiagnostic variables documented in previous studies, use of externalizing psychopathologies, use of comprehensive multivariate statistical analysis, and whether there was a discussion about the scope of a transdiagnostic model and the relevance for a systematic review.

### 2.7. Summary of Results

We presented the results in a selection and inclusion graph of studies according to the PRISMA model, in addition to a subsequent table showing the different studies included in the systematic review, according to the data extraction plan. The findings were grouped according to structural, emotional, and cognitive levels with variables related to the behavioral activation and inhibition system, emotional dysregulation, motivation, and cognition, as a narrative synthesis.

## 3. Results

Figure 1 illustrates the process of identifying and selecting the 19 studies that were included in this systematic review according to the PRISMA model.

### 3.1. Characteristics of the Studies

The studies evaluated corresponded to antisocial psychopathologies according to the age specifications and transdiagnostic variable of interest. We grouped the 19 studies included in the systematic review into eight focused on externalizing symptoms (aggressiveness and irritability) (42.1%), six from the spectrum of conduct disorder (31.6%), two on psychopathy (10.5%), one on substance abuse -alcohol- (5.3%), and two generics without reporting any disorder (10.5%), but which met the established eligibility criteria. A summary according to the synthesis of results foreseen in the method is summarized in Table 2, Table 3 and Table 4.

The reviewed studies fulfill the objectives of the research because they report AD in the reference population. They also fit the age specifications defined for the study and, in turn, indicate possible transdiagnostic variables for these problems. To analyze the information collected, we decided to organize each study according to the results synthesis plan. According to the behavioral activation and inhibition system, we found seven studies (36.84%) (Table 2), and 12 studies related to emotional regulation, motivation and cognition (63.16%) (Table 3 and Table 4).

### 3.2. Assessment of the Methodological and Theoretical Quality

We established the quality of the selected studies according to the assumptions of the DIAD model [20]. This procedure was adapted reviewing if the following methodological aspects were met in each document, with the coding of yes = 1 point, no = 0 points, according to the methodological characteristics and quality of the included studies: Study Type (ST) if it was a quantitative study (1 point), associative -correlational or comparative- (1 point), and multivariate (1 point) or interventional (1 point): total 3 points, in case study: 1 point); in Sample (SS), if it was an adequate sample size (includes sample calculation): 1 point); in the Participant Selection (PS), if it included an adequate description of participant selection (Adolescents and Youth): 1 point); if it had reliable and valid instruments (RVI, 1 point); if it showed transdiagnostic variables documented in previous studies (TV, 1 point); if the study analyzed the externalizing psychopathologies (EP, 1 point); if it reported Multivariate Statistical Analysis (MSA, 1 point); in addition, we verified if the scope of an externalizing transdiagnostic model was discussed (TMD, 1 point).

A value of the total methodological and transdiagnostic relevance for systematic review (TMTR, 0–5, Low relevance for the study -lees than 50% of quality characteristics-; 6–8, Mean relevance for the study -between 60% and 80% of quality characteristics-; 9–0, High relevance for the study -between 90% and 100% of quality characteristics-) was estimated to establish the quality of the study. In general terms, we rated the relevance for the study at a methodological level; for the development of the systematic review, we estimated five low quality (26.32%), eight medium quality (42.11%), and six high quality (31.57%) articles. The summary of the studies that we reviewed for methodological quality is shown in Table 5.

In the assessment by type of study, we found that most were multivariate with correlational or comparative analysis, 68.42% (*n* = 13) met the criteria, followed by 26.32% (*n* = 5) with two points, and one study (5.26%) with one point for being quantitative. In terms of sample size, 89.47% (*n* = 17) did not submit the sample size calculation; likewise, in the description of the selection of participants (adolescents and youth), 78.95% (*n* = 15) did so appropriately. For measuring instruments, all studies presented the reliability and validity indicators (100%, *n* = 100).

On the other hand, this study found that 78.95% (*n* = 15) included the use of transdiagnostic variables documented in previous studies, compared to 21.05% (*n* = 4) that included other possibly transdiagnostic variables [4]; likewise, in the use of externalizing psychopathologies, 52.63% (*n* = 10) the study reported them compared to 47.37% (*n* = 9) that did not specify them in the method. Also, we checked whether the studies used multivariate statistical analysis, and found that 89.47% (*n* = 17) reported it. Finally, we assessed whether there was any discussion about the scope of a transdiagnostic model and whether it was relevant for inclusion in the systematic review. We found that 42.11% (*n* = 8) did so explicitly and associated the analysis directly with the TM.

### 3.3. Qualitative Synthesis

We reviewed the transdiagnostic variables in antisocial psychopathologies of adolescents and young adults, within the framework of advances in behavioral activation and inhibition systems, anger, aggressiveness, motivation, emotional dysregulation, and cognition. Mainly in oppositional behavior disorders, aggressiveness, ADHD, Externalizing problems, and alcohol abuse.

The findings made in this study reported evidence of transdiagnostic models composed of higher-order factors in terms of externalizing psychopathology [22,23,25,26], negative affectivity as distinctive trait [24,25,27], and impulsivity related to sensation-seeking [21,26,30]. Variables that are constituted as evidence of a transdiagnostic structural model of AD. Other studies highlight emotional irritability [28], and difficulty regulating of emotions as the main predictors of AD. Also, these findings reported a high correlation and prediction coefficients (*r* and *β*) [32,33,34,35], added to the impairments in emotion recognition [29], and callous-unemotional [29,30,31]. Finally, studies that highlight the role of perceptual-cognitive biases [36], associated with violent ideation [38], and anger rumination as a key predictor of AD (a variable that has been suggested to be analyzed as a second-order factor [37,39]) were found at the cognitive level.

## 4. Discussion

The study aimed to identify through a systematic review, the main transdiagnostic variables in antisocial disorders of adolescence and youth. Given these findings, studies were reported in which there is evidence of models according to structural, emotional, and cognitive transdiagnostic variables.

### 4.1. Personality, Inhibition-Behavioral Activation and Negative Affect

In this study, evidence of the investigation regarding the general externalizing factor (P factor) was found, which is a latent psychopathological lability in the disorders reported in samples of pre-adolescents and adolescents. This P factor is associated with low active control and a high negative affect. However, researchers question this last aspect, arguing that only in the externalizing symptoms there was clear evidence of low active control [25], especially in male samples who present greater externalizing symptoms and require treatments focused on reducing impulsivity and sensation-seeking (high Behavioral Activation System—BAS—and low Behavioral Inhibition System —BIS) [21].

However, the one-factor model has been questioned due to the appearance of a correlated two-factor proposal, in which it seems that psychopathologies share both internalizing and externalizing aspects. In this sense, it was found a study that reports a two-factor personality model composed of an externalizing and an internalizing one. This model was composed of a general factor of high disinhibition, impulsivity, neuroticism, low affability, and intelligence. In turn, associated with an externalizing factor composed of substance consumption, sensation seeking, extraversion, and attentional biases towards negative-valence stimuli [22]. A model that could be consolidated as a higher-order model, an indicator of the severity of a higher-order personality disorder (hierarchical TM) that has been associated with a personality disorder, aggression and negative affect as a trait [36]; these hierarchical models have demonstrated important indicators of predictive validity in a variety of both internalizing and externalizing disorders, as well as comorbidities [40], many of which are associated with oppositional defiant disorders in a higher-order latent factor [41], accompanied by interpersonal problems, substance abuse, and psychosocial impairment [42].

On the other hand, components related to inhibition of aggressive behavior (high BAS and low BIS), can be evidenced in responsiveness to emotion, which seems to be associated with the appearance of externalizing symptoms of anger, hostility, and aggression even in internalizing ones such as anxiety and suicidal risk, although not in alcoholism, results that suggest further research on responsiveness to emotion [30]. Similarly, one of the transdiagnostic variables identified was irritability as a tonic trait, which is associated with a 71.3% increase in disruptive problems in adolescents and young people over a one-year follow-up period. However, it decreases with age due to increased behavioral control [28], while internalizing symptoms increase [43], regardless of sex, at least in adolescents and young people with differential levels of disruptive functioning [28]. However, these findings need to be further deepened with more empirical evidence, given the low methodological quality criteria obtained for these studies [28,30,36].

These results highlight the role played by experiential avoidance as a behavioral inhibitor. It was reported that this variable had a low predictive capacity in externalizing symptoms by explaining 8.3% of the variance (*r* = 0.36), in addition to its relationship with peer victimization (relational aggression) [34]. An aspect that could indicate that experiential avoidance as a disadaptive behavior that appears in externalizing psychopathologies in the face of discomfort (negative affect) as a motivational element from a contextual model [44]. However, the study was conducted as a small sample composed 100% of urban women and ethnic minorities, in which they claim that experiential avoidance is an avoidance behavior associated with internalizing psychopathologies [34], which also highlights the low methodological quality of this study obtained with the DIAD model [34]. In general terms, experiential avoidance as a transdiagnostic variable is viable for an explanatory model of AD, given that it has presented associations with physical, psychological and sexual aggression [45], behavioral problems, substance abuse and sexual promiscuity [46].

In this regard, inhibitory mechanisms play a fundamental role in increasing the vulnerability of AD, especially when they are composed of cognitive (distress, control, and coping) and affective (positive and negative affect) reactions. In the case of reactions in the face of daily stressors, these are related to the internalizing spectrum and, to a lesser extent, to the externalizing ones due to their emotional nature, which is not very inhibiting, may achieve a decrease in their negative affect response [27]. Therefore, in the occurrence of aggressive or other related behaviors, responses related to difficulties in inhibitory control and parasympathetic regulation at early ages [47], although they increase in adolescence according to prospective longitudinal reports [48].

Besides, behavioral variations and psychopathic characteristics present in late adolescent and young adult offenders require more precise monitoring, in addition to taking into account variations between genders, given those social relationship problems with peers and family members, increased violence and cruelty to others, and even the assumption of adult roles have been reported [26] (Table 2).

### 4.2. Emotional Dysregulation, Motivation and Cognition

Anger in AD has been a permanent focus of interest for research in psychopathology. It is a common predisposition factor for reactive aggression in externalizing disorders, a key point for the appearance of different symptoms, including internalizing disorders such as depression and post-traumatic stress [49]. The processes involved include selective attention, memory bias, distorted reasoning, negative rumination [50], in addition to the basic executive function for effective emotional regulation [49].

In this way, anger dysregulation as an externalizing transdiagnostic variable stands out as part of a TM of AD. It has been reported that adolescents with high DE are at greater risk of substance abuse and the development of externalizing and internalizing symptoms [51]. DE is associated with predispositions to aggressive behavior and reduced processing of inhibitory control [52]; for this reason, DE has been considered a key variable for the prevention of externalizing behavior problems, mainly if strategies involving executive control, decision-making and coping are used [53].

In samples with adolescents with a high DE profile (low tolerance to frustration, impatience, immediate anger, rapid arousal by emotional reactions), in isolation, social problems, rule-breaking, and somatic complaints at the CBCL scored higher; as well as additional high scores on scales such as narcissism and impulsivity, but not emotional distress, results that are indicative of vulnerability to the development of secondary psychopathic traits [31]. Adolescents in a longitudinal study who were measured over seven months, they found that DE at time 1 predicted aggression at time 2 (*β* = 0.18, *p* < 0.001), however, psychopathology at time 1 did not predict an elevated DE at time 2, these data provide evidence for the causal direction of this transdiagnostic variable in adolescent psychopathology [32].

This variable is associated with different alterations; for example, increases in cortisol in people with DE have been reported to predict externalizing symptoms such as oppositional defiance (*β* = −0.74, *p* < 0.001) and ADHD (*β* = −1.15, *p* < 0.001), and substance use (*β* = −0.36, *p* < 0.001), although with low effect sizes [33]. Another alteration has been a positive and negative affect as a trait since it increases the use of emotional regulation strategies at the interpersonal level in a negative way (externalizing problems). Also, when people have a low positive affect trait are at greater risk of generating interpersonal problems by decreasing functional interaction with others; in addition to the increase in the pattern of avoidance and the deterioration of interpersonal relationships, which leads to opportunities for learning skills that regulate emotional responses in adolescents and youth [24].

In this line, even in patients with autism, DE in recognition of emotions, in cases with high emotional callus, training in this skill has generated increased empathic response and therefore decreased externalizing symptoms [29], a favorable indicator of this transdiagnostic variable in this population. Thus, lack of empathy is related to DE in a possible TM, in that difficulties associated with deficits in emotional regulation with cognitive processes involved as altered processing in emotional recognition during regulation of anger responses, directly associated with aggressive, alexithymic responses and poor empathy, evidence reported in people with intermittent explosive problems [35]; these new hypotheses should be reviewed in studies covering a much wider range of AS.

In summary, DE could be constituted as a transversal factor in internalizing and externalizing psychopathologies. It has been suggested that DE in AD in adolescents and young adults that (a) at the subcortical level presents a mediating role of impulsivity as a temperamental characteristic (trait), a key aspect in the socialization capacity in different disorders (even internalizing ones such as anxiety), (b) is acquired and maintained by negative reinforcement on repeated occasions of stress in dysfunctional families and in interaction with peers, (c) this reinforcement of neuronal plasticity at these ages alters the functioning of the prefrontal cortex resulting in poor executive control (top-down) and altered patterns of vagal response to emotion recall [54] (Table 3).

On the other hand, as for traditional models of cognition, it has been found that perceptual biases in the face of stressors or emotional events predict both internalizing and externalizing traits. These biases are aimed at decreasing the impact of stressors and therefore increase externalizing symptoms such as antisocial behavior and substance abuse, which is associated with a hyposensitivity to stressors that increase symptomatic levels of the externalizing spectrum [23] (contributions that should be verified with studies with a higher methodological quality given their low DIAD score). Subsequently, it was reported that threat interpretation biases, low social support and poor active coping were associated with externalizing symptoms [55]; also, in a meta-analysis of 212 studies, the main factors related to higher externalizing and internalizing symptoms were ED (avoidance, emotional suppression, and denial) and primary and secondary maladaptive cognitive coping (interpretative biases and control behaviors) [56].

Rumination was another cognitive variable. Its predictive and mediating capacity was documented in a transdiagnostic model of symptoms such as aggression, anxiety, and aggressive behavior. For example, in a three-measure, seven-month longitudinal study, it was found to constitute a mechanism for explaining the onset of internalizing symptoms and subsequent aggressive behavior in males, which appears to underlie the transition from aggressive behavior to anxiety, but also to explain the onset of aggressive behavior in adolescents with anxiety and depression [37]. It can be stated that anger rumination is constituted as a set of cognitive abilities consisting of thoughts that function in a second-order, which would allow us to explain the overlaps that appear in different psychopathologies, particularly, in executive functions in externalizing disorders, although this evidence suggests that it is not possible to support causal hypotheses yet [39].

In turn, rumination is closely associated at the cognitive level with another potentially transdiagnostic variable, such as violent ideation. It has presented significant associations with different mental health problems such as reactive, physical and proactive aggression [33], especially as a predictor in cases of poor self-control; however, it is not yet clear if violent ideation reflects the aggressive manifestation and not a causal variable [57] (Table 4). However, studies are inconclusive and require differentiation in terms of negative content and anger, particularly when closely linked to different psychopathologies, violent acts, and a general deterioration of mental health [58].

### 4.3. Limitations

This systematic review had limitations that need to be improved in further studies. The first was not having included other types of refereed documents, which do not appear in the consulted databases (theses, books, etc.), or those written in languages other than English. The second limitation was the non-inclusion of studies focused on treatments of antisocial psychopathology, given that in the analysis of the effects of these interventions, transdiagnostic variables of interest could appear. We recommend for future reviews to include psychotherapies that include the variables found and to perform a meta-analysis with these reports. Finally, a third limitation corresponded to the studies that contained transdiagnostic analyses with internalizing and externalizing psychopathologies, which could generate partial evidence for the study of antisocial psychopathology. In future reviews, it is recommended to propose as exclusion criteria, the simultaneous internalizing problems in the selected studies.

### 4.4. Clinical Implications

The clinical implications of this study are the development of new directions of a transdiagnostic model in which the identified variables are submitted together. The approach should maintain the transdiagnostic approach of the multiple processes, that is, the search for the least number of key mechanisms with the greatest explanatory power for the understanding of comorbid disorders [6]. Also, having a transdiagnostic model that has been empirically reviewed will solve the limited progress made in this field by following a position that allows for the integration of various levels of analysis of theoretical and empirical knowledge that will have an impact on efforts to promote and prevent these problems [8]. Ultimately, this will favor the development of temporarily less costly treatments, and thus will allow for the promotion of prevention alternatives in directions other than those currently available.

## 5. Conclusions

Advances are made for the consolidation of a transdiagnosis model framed in the guidelines of the RDoC project for externalizing psychopathologies in adolescents and young people. Within the framework of a general psychopathological factor, in the case of an AD model, this is a general psychopathic or externalizing factor, in which the variables are integrated as fundamental structures of the inhibition and activation systems and negative affect as a trait. There is more evidence for these variables, and they may be associated with subsequent experiential avoidance, ED, anger rumination, and violent ideation.

It is necessary to develop new directions around the development of a transdiagnostic model for AD. Firstly, measures of change must be incorporated through longitudinal studies, either with a model of developmental trajectories or models that allow a prospective analysis taking into account the variables reported in this review, since this type of psychopathology, variations in behavior, contexts and other variables such as gender, can generate limitations in the development of a complete model of these problems.

## Figures and Tables

**Table 1:** Reviewed and descriptors Used in the Meta-searchers Consulted.

Databases	Descriptors
Science Direct	Transdiagnostic and antisocial
Proquest	Transdiagnostic and aggression
Health & Medical Collection	Transdiagnostic and delinquency
Medline	Transdiagnostic and violence
Nursing and Allied health database	Transdiagnostic and anger
PsycArticles	Transdiagnostic and psychopathy
PsycBooks	Transdiagnostic and antisocial personality disorder
Psychology Database	Transdiagnostic and conduct disorder
PsycInfo	Transdiagnostic and dissocial disorder
PsycTest	Transdiagnostic and externalizing
Psicodoc	

Source: Authors.

**Table 2:** Summary of reviewed empirical studies including, transdiagnostic variables in antisocial disorders at the structural level.

Authors	Country	N	Age in Years M(SD)	Male and Female %	AD Assessed	Measure	Transdiagnostic Variable	Key Findings
Carragher et al. [21]	Australia	2175	13.3 (0.48)	M = 57.4% F = 42.6%	Externalizing symptoms (behavior and hiperactivity), alcohol consumption	Strengths and Difficulties Questionnaire (SDQ), Diagnostic Interview Schedule for Children	Impulsivity, Sensation seeking	They tested a model with a general psychopathology factor. Men show more externalizing symptoms. They point out that adolescents require treatments focused on decreasing impulsivity, and sensation-seeking.
Castellanos-Ryan et al. [22]	Canada	2144	14.39 (0.77)	M = 49% F = 51%	Conduct Disorder (CD), Attention-Deficit/Hyperactivity Disorder (ADHD), Oppositional-Defiant Disorder (ODD)	Development and Well-Being Assessment interview (DAWBA), Alcohol Use Disorders Identification Test (AUDIT), Substance Use Risk Profile Scale (SURPS), NEO Five Factor Inventory (NEO FFI), Temperament and Character Inventory (TCI), Wechsler Intelligence Scale for Children—4th edition (WISC-IV), Test Automated Battery (CANTAB), Spatial Working Memory (SWM).	Externalizing personality factor	They present a two-factor personality model composed of an externalizing, and an internalizing one. The externalizing factor is associated with substance consumption, sensation-seeking, extraversion, and attentional biases to negative valence stimuli. The general factor is characterized by high disinhibition, impulsiveness, neuroticism, low affability, and performance when measuring intelligence.
Conway et al. [23]	Australia	700	20 (NR)	M = 48.28% F = 51.72%	Personality Pathology PD Externalizing diagnosis (substance use and disruptive behavior disorder)	Structured Clinical Interview for DSM-IV (SCID), Structured Clinical Interview for DSM-IV Axis II Personality Disorders, Version 2.0 (SCID-II), Longitudinal Interval Follow-Up Examination (LIFE), Aggression, and Delinquency subscales of the Young Adult Self Report (YASR)	Externalizing personality factor	A two-factor model appears with a higher severity factor of the personality disorder (hierarchical MT). Significant correlations between personality disorder, aggression, and negative affect as a trait (both with r = 0.46)
Hamilton et al. [24]	USA	304	18.20 (1.39)	M = 32% F = 68%	Externalizing problems	Behavioral Inhibition System/Behavioral Activation System Scales (BIS/BAS), Sensitivity to Punishment/Sensitivity to Reward Questionnaire (SPSRQ), Positive and Negative Affect Schedule (PANAS), Responses to Positive Affect Scale (RPAS), Life Events Scale (LES) and Life Events Interview (LEI), Beck Depression Inventory (BDI).	Positive and negative trait affect and emotion regulatory	Positive and negative affect as a trait is associated with an increase in emotional regulation strategies in positive and negative interpersonal events (externalizing problems). People with high negative affect traits would be at greater risk of increasing depressive symptoms, while in low positive affect traits, they would be at greater risk of interpersonal problems (people who decrease their positive responses decrease their functional interaction with others).
Hankin et al. [25]	USA	571	13.58 (2.37)	M = 44.5% F = 55.5%	Oppositional Defiant Disorder or Conduct Disorder (ODD/CD)	Children’s Depression Inventory (CDI), Manifest Anxiety Scale for Children (MASC), Child Behavior Checklist (CBCL/YSR), Aggression scale of the Early Adolescent Temperament Questionnaire Revised (EATQ-R), Swanson, Nolan, and Pelham scale (MTA SNAPIV), Positive and Negative Affect Scale for Children (PANAS-C).	Temperament as a dimensional and latent psychopathological factor Positive and Negative Affect. Active control (effortful control).	Latent psychopathological lability was found in the pathologies (P Factor), both in the pre-adolescent and adolescent samples. The P factor was associated with low active control and high negative affect. In internalizing problems, a high negative and low positive affect was associated with the P factor, while in the externalizing problems, only a low active control was associated.
Hawes et al. [26]	USA	1170	14–17	M = 100% F = 0%	Psychopathy Alcohol use	Index of Social Position (ISP), Youth Psychopathic Traits Inventory-Short Version (YPI-S), Substance Use/Abuse Inventory (Quantity and Frequency Index, QFI), Proportion of supervised time (PST)	Psychopathic trait factor (Interpersonal, Callous-Unemotional, Impulsive-Irresponsible)	Variations were found in the change rates of late adolescent and young adult offenders, in terms of alcohol use and psychopathic characteristics (social relationship problems with peers and family, assuming adult roles, increased violence, and cruelty to others), especially the year following the start of consumption.
South et al. [27]	USA	78	20.8 (2.23)	M = 29% F = 71%	High order externalizing factor: Aggression factor and Substance use factor	Inventory of Depression and Anxiety Symptoms (IDAS), Personality-based Externalizing Scale (EXT-P)	Positive-Negative Affect.	Cognitive (Distress, Control, and Coping) and affective (Positive and Negative Affect) reactions to everyday stressors are related to the internalizing spectrum and less to the externalizing spectrum, due to its low inhibitory emotional nature. They add that externalizing measures moderate the association between Distress and negative affect. People who are high in externalizing problems and have a high sense of control over their environment decrease their negative affect response.

Note: N (sample size), M (male), F (female), AD (antissocial disorder). Source Authors.

**Table 3:** Reviewed empirical studies including transdiagnostic variables in antisocial disorders at an emotional level.

Authors	Country	N	Age in Years M(SD)	Male and Female %	AD Asessed	Measure	Transdiagnostic Variable	Key Findings
Copeland et al. [28]	USA	1420	9 to 16 (cohort: 9, 11, 13, 16)	M = 51% F = 49%	No AD was reported.	Child and Adolescent Psychiatric Assessment (CAPA), Child and Adolescent Burden Assessment	Tonic and phasic irritability as a transdiagnostic marker	Irritability decreases with age. Irritability does not vary between genders, nor is it associated with an increased risk of disruptive problems. It had little to do with a common profile of disruptive functioning in different psychiatric disorders.
Dadds et al. [29]	Australia	195	10.52 (2.64)	M = 75.89% F = 24.11%	Oppositional Defiant Disorder or Conduct Disorder (ODD/CD), Predominately inattentive Attention Deficit Disorder or combined Attention DeficitHyperactivity Disorder (ADD and ADHD)	Child Adjustment on the Strengths and Difficulties Questionnaire (SDQ), Family and Child Experiences Survey (FACES), Griffith Empathy Measure (GEM)	Recognition of emotions as part of the transdiagnostic treatment based on the Emotional Regulation Trait: Emotional callus	Training in recognition of emotions had little impact on behavioral problems. Training in emotion recognition has a favorable impact on children on the autism spectrum. Children with high emotional callus showed little response to training in emotion recognition, although the empathic response increased.
Jonhson et al. [30]	USA	312	18.77 (1.90)	M = 31% F = 69%	Externalizing symptoms of anger, hostility and physical aggression. Alcohol Use Disorder.	Aggression Questionnaire short Form (AQ), Mood and Anxiety Symptoms Questionnaire -Somatic Arousal subscale (MASQ-SA), Alcohol Use Disorder Identification Test (AUDIT), McLean Screening Instrument for Borderline Personality. Disorder (MSI-BPD)	Impulsive Reactivity (negative generalization, impulsive tendency, positive urgency, persistent lack of self-control, low scrupulosity, low reflective reaction)	Reactivity to emotion correlates with the externalizing symptoms of anger, hostility, and aggression, in the internalizing type with anxiety. The authors emphasize that emotional regulation is focused on decreasing impulsivity in the psychopathologies of both spectra. No association with alcoholism was found.
Masi et al. [31]	Italy	108	9–15 years ODD 10.37 (1.8) CD 10.27 (1.9)	M = 83.33% F = 16.66%	Oppositional Defiant Disorder and Conduct Disorder (ODD/CD)	Schedule of Affective Disorders and Schizophrenia for School-Age Children Present and Lifetime Version (K-SADS-PL), Child Behavior Checklist (CBCL), Antisocial Process Screening. Device Parent version (APSD), Alabama Parenting. Questionnaire (APQ), Clinical Global Impression Severity (CGI-S), Children’s Global Assessment Scale (C-GAS).	Emotional dysregulation. Emotional callouses.	Participants with an Emotional dysregulation profile (low tolerance to frustration, impatience, immediate anger, rapid arousal by emotional reactions) scored higher on isolation, social problems, rule-breaking, and somatic complaints at CBCL. Higher scores appeared for narcissism and impulsivity, though not for emotional callouses.
Mclaughlin et al. [32]	USA	1065	11–14	M = 51.2% F = 48.8%	Aggressive behavior	Children’s Depression Inventory (CDI), Multidimensional Anxiety Scale for Children (MASC), Revised Peer Experiences Questionnaire (RPEQ), Children’s Eating Attitudes Test (ChEAT), Emotion Expression Scale for Children (EESC), Children’s Sadness Management Scale (CSMS), Anger Management Scale (CAMS), Children’s Response Styles Questionnaire (CRSQ).	Emotional dysregulation	They found that emotional dysregulation predicted aggression (β = 0.18, *p* < 0.001). In turn, psychopathology does not predict the appearance of emotional dysregulation; this provides evidence on the causal direction of this transdiagnostic variable in adolescent psychopathology.
Poon et al. [33]	USA	134	12–14 years 12.61 (0.50)	M = 55% F = 45%	Substance abuse, Conduct Disorder (CD), Attention-Deficit/ Hyperactivity Disorder (ADHD), Oppositional-Defiant Disorder (ODD)	Salivary cortisol levels, Difficulties in Emotion Regulation Scale (DERS), Youth Risk Behavior Survey questionnaire (YRBS), Children’s Depression Inventory (CDI), Revised Children’s Manifest Anxiety Scale (RCMAS), Child Symptom Inventory (CSI)	Emotional dysregulation	Difficulties in emotional regulation and high cortisol predict substance abuse behaviors (β = −0.36, *p* < 0.001), although with a small effect size (OR = 0.70). These difficulties also predict the appearance of externalizing symptoms, in ODD (β = −0.74, *p* < 0.001), and ADHD (β = −1.15, *p* < 0.001), in CD or ADHD was not significant.
Shea et al. [34]	USA	58	12.26 (0.98)	F = 100%	Externalizing disorders (Aggressive Behavior and Rule-Breaking Behavior)	Revised-Social Experiences Questionnaire-Self Report (SEQ), Child Behavior Checklist—Youth Self-Report (YSR), Avoidance and Fusion Questionnaire for Youth (AFQ-Y)	Experiential avoidance	Experiential avoidance is related to the victimization of relational peer aggression. It explains 8.3% of the variance in the externalizing problems (r = 0.36). These data indicate that experiential avoidance increases the risk of victimization in adolescent women. The study found more evidence for internalizing disorders (34.3% of variance); the authors suggest that this may be a form of avoidance behavior.
Fahlgren et al. [35]	USA	492	18–55 M = 25.97(9.57)	F = 66.86% M = 33.14	Intermittent explosive disorder	Structured diagnostic interview for DSM-IV (SCID-I), Structured interview for DSM-IV personality disorders (SIDP), IED interview (IED-I), Sadness and anger rumination inventory (SARI), Ruminative response scale (RRS-10), Toronto alexithymia scale (TAS-20), Reading the mind in the eyes task (RMET), Basic empathy scale (BES), Quality of life enjoyment and satisfaction questionnaire – short form (Q-LES-Q-SF), Life history of aggression interview (LHA)	Emotional dysregulation and anger-rumination	Intermittent explosive problems are associated with deficits in the regulation of emotions, with cognitive processes involved as altered processing in recognition of emotion, which is associated with aggressive, alexithymic responses and difficulty in empathy in recognition of emotions.

Note: N (sample size), M (male), F (female), AD (antissocial disorder). Source Authors.

**Table 4:** Reviewed empirical studies including transdiagnostic variables in antisocial disorders at the cognitive level.

Authors	Country	N	Age in Years M(SD)	Male and Female %	AD Assessed	Measure	Transdiagnostic Variable	Key Findings
Conway et al. [36]	Australia	705	20	M = 86.5% F = 13.5%	Alcohol abuse/dependence Drug abuse/dependence	Structured Clinical Interview for DSM-IV (SCID), Structured Clinical Interview for DSM-IV Axis II Personality Disorders, Version 2.0 (SCID-II), Longitudinal Interval Follow-Up Examination (LIFE), Aggression, and Delinquency subscales of the Young Adult Self Report (YASR)	Perceptual biases: Cognitive Risk Marker	Perceptual biases, in response to stressors or emotional events, predict both internalizing and externalizing traits. Hyposensitivity to stressors increases the levels of the externalizing spectrum. Biases, aimed at decreasing the impact of stressor, increase externalizing symptoms (antisocial behavior and substance abuse).
Mclaughlin et al. [37]	USA	1065	11–14	M = 51.2% F = 48.8%	Aggressive behavior	Children’s Response Styles Questionnaire (CRSQ), Peer Experiences. Questionnaire (RPEQ), Children’s Depression Inventory (CDI), Multidimensional Anxiety Scale for Children (MASC).	Rumination	The study found that rumination is a mechanism to explain the appearance of internalizing symptoms and subsequent aggressive behavior in males. Rumination predicts aggressive behavior and vice versa. Thus, rumination underlies the transition from aggressive behavior to anxiety, but it also explains the emergence of aggressive behavior in adolescents with anxiety and depression.
Murray et al. [38]	Switzerland	1306	17	M = 50.45% F = 49.55%	Aggressive behavior, Attention deficit hyperactivity disorder (ADHD)	Violent Ideations Scale (VIS), Social Behaviour Questionnaire (SBQ)	Violent ideation (thoughts of killing, hitting, intimidation, causing pain and humiliation)	Violent ideations had moderate correlations with ADHD, proactive/indirect aggression, and reactive/physical aggression. However, the ADHD measure was the only one that did not correlate with mental health measures. The authors stress that they do not appear to be directly associated with the other externalizing problems and should, therefore, be considered a marker for mental health difficulties.
du Pont et al. [39]	USA	764	22.8 (1.3)	M = 47.38% F = 52.62%	Externalizing Psychopathology	Rumination-Reflection Questionnaire (RRQ), Anger Rumination Scale (ARS), and nine EF laboratory tasks; Diagnostic Interview Schedule–IV (DIS-IV), Composite International Diagnostic Interview–Substance Abuse Module (CIDI-SAM).	Anger rumination, depressive rumination, inhibition, and executive function	Anger rumination is a set of cognitive skills represented in second-order thoughts that allow explaining the overlaps in the different psychopathologies, mainly the executive functions in externalizing disorders.

Note: N (sample size), M (male), F (female), AD (antissocial disorder). Source Authors.

**Table 5:** Summary of the methodological and theoretical quality of the empirical studies reviewed that include transdiagnostic variables in antisocial disorders (*n* = 19).

Studies	ST	SS	PS	RVI	TV	EP	MSA	TMD	TMTR	Quality
Carragher et al. [21]	3	No	No	Yes	No	Yes	Yes	Yes	7	Mean
Castellanos-Ryan et al. [22]	3	No	Yes	Yes	No	Yes	Yes	No	7	Mean
Conway et al. [36]	2	No	No	Yes	No	No	Yes	No	4	Low
Conway et al. [23]	3	No	No	Yes	No	No	Yes	No	5	Low
Copeland et al. [28]	2	No	Yes	Yes	Yes	No	No	No	5	Low
Dadds et al. [29]	3	No	Yes	Yes	Yes	Yes	Yes	No	8	Mean
Hamilton et al. [24]	3	Yes	Yes	Yes	Yes	No	Yes	Yes	9	High
Hankin et al. [25]	3	No	Yes	Yes	Yes	Yes	Yes	Yes	9	High
Hawes et al. [26]	3	No	Yes	Yes	Yes	Yes	Yes	Yes	9	High
Jonhson et al. [30]	2	No	No	Yes	Yes	No	Yes	No	5	Low
Fahlgren et al. [35]	3	No	Yes	Yes	Yes	Yes	Yes	Yes	9	High
Masi et al. [31]	1	No	Yes	Yes	Yes	Yes	Yes	Yes	7	Mean
Mclaughlin et al. [32]	3	No	Yes	Yes	Yes	No	Yes	No	7	Mean
Mclaughlin et al. [37]	3	No	Yes	Yes	Yes	No	Yes	No	7	Mean
Murray et al. [38]	3	Yes	Yes	Yes	Yes	Yes	Yes	No	9	High
Poon et al. [33]	2	No	Yes	Yes	Yes	Yes	Yes	No	7	Mean
Shea et al. [34]	2	No	Yes	Yes	Yes	No	No	No	5	Low
South et al. [27]	3	No	Yes	Yes	Yes	No	Yes	Yes	8	Mean
du Pont et al. [39]	3	No	Yes	Yes	Yes	Yes	Yes	Yes	9	High

Note: source authors.
